# Routine primary care data for scientific research, quality of care programs and educational purposes: the Julius General Practitioners’ Network (JGPN)

**DOI:** 10.1186/s12913-018-3528-5

**Published:** 2018-09-25

**Authors:** Hugo M. Smeets, Marlous F. Kortekaas, Frans H. Rutten, Michiel L. Bots, Willem van der Kraan, Gerard Daggelders, Hanneke Smits-Pelser, Charles W. Helsper, Arno W. Hoes, Niek J. de Wit

**Affiliations:** 1 Julius Centre for Health Sciences and Primary Care, University Medical Centre Utrecht, P.O. Box 85500, 3508 GA Utrecht, The Netherlands; 2 General practice De Grebbe, Rhenen, The Netherlands; 3 General practice Mariahoek aan de Singel, Utrecht, The Netherlands; 4 General practice Health Centre De Bilt, De Bilt, The Netherlands; 5 General practice Health Centres Leidsche Rijn, Utrecht, The Netherlands

**Keywords:** Electronic medical record, Quality of care, Database, Research, ICPC

## Abstract

**Background:**

General Practitioners (GPs) in the Netherlands routinely register all patient contacts electronically. These records include longitudinally gathered clinical information of the patient contacts in coded data and free text.

**Methods:**

Diagnoses are coded according to the International Coding of Primary Care (ICPC). Drug prescriptions are labelled with the Anatomical Therapeutic Chemical Classification (ATC), and letters of hospital specialists and paramedic health care professionals are linked or directly incorporated in the electronic medical files. A network of a large group of GPs collecting routine care data on an ongoing basis can be used for answering various research questions.

**Results:**

The Julius General Practitioners’ Network (JGPN) database consists of routine care data from over ten years of a dynamic cohort of around 370,000 individuals registered with the participating GPs from the city of Utrecht and its vicinity. Health care data are extracted anonymously every quartile of a year and these data are used by researchers.

**Conclusion:**

We describe the content and usability of our JGPN database, and how a wide variety of research questions could be answered, as illustrated with examples of published articles.

## Background

The Dutch health system is based upon a gatekeeper system, in which patients only have access to hospital care after consulting the general practitioner (GP). All Dutch residents are registered with a GP, and all have mandatory health care insurance. GPs provide basic care for all health problems for all patient categories; emergency care, chronic disease, and mental health problems. GP care is completely insured and is not subject to initial payment; therefore the threshold for consultation is low. GP registries therefore adequately reflect morbidity patterns of the Dutch population, as far as it results in a contact with a healthcare provider.

General Practice has a long-standing history of registering routine healthcare data. In the early days of general practice, some GPs in the UK presented joint patients records as practice overviews to study morbidity patterns in primary care [1, 2]. In the Netherlands Huygen kept a very detailed registration of patient contacts and demonstrated that such registration of routine care data could be used to answer clinically relevant research questions [3]. Over the last three decades electronic patient records have increasingly replaced traditional paper files. With the introduction of electronic General Practice Information Systems (GPIS), patient contacts could be studied more easily [4]. Initially, registration was primarily done for practice archiving, and data quality was variable. Later, professional organizations such as the Royal College of GPs in the UK, and the Dutch College of GPs (NHG) stimulated consistency and reproducibility of coding by the introduction of the structured diagnostic coding systems: READ codes in the UK, and International Classification of Disease Codes (ICPC) in the Netherlands [5, 6]. Large-scale use of these coding systems improved registration uniformity and made routine care data accessible and attractive for research [7]. The READ diagnostic coding system provided more detailed diagnostic information than the ICPC-coding system, enabling more diagnostic details for research. In more recent years, routine care databases also proved suitable for the monitoring of quality of care in general practice. In the UK, GPs within the National Health Service (NHS) monitor relevant quality parameters with standardized templates as part of the routine clinical process. In the Netherlands, GP network organisations initiated coding and monitoring of chronic disease management, which further improved the quality of the records in terms of completeness and accuracy [8].

Routine care databases are widely used for research. Primary care research networks originated from local initiatives, but in recent years they merged to large-scale registration databases in many countries [4, 9–11]. In the UK, the General Practice Research Database (GPRD) has been shown to be an effective environment for large scale observational research in primary care. Catalonia (SIDIAP), Scotland (ESCRO), and Canada (CPSSN) also provide comparable databases enabling research.

Academic research in routine primary care databases in the Netherlands has a longstanding tradition. In the 1960s a number of dedicated general practitioners in the Nijmegen area started the Continuous Morbidity Registration (CMR), which at present has more than 40 years of follow-up registration data of 15,000 patients [12, 13]. In 1970 in the Amsterdam region, Lamberts introduced the ICPC coding system in the Netherlands, initiated in ‘the Transition project’; reasons for encounter and (working) diagnoses were uniformly coded [14–16]. In 1998, The Netherlands Institute for Primary Care (Nivel) conducted the first national survey of general practices, resulting in a countrywide registry of presented morbidity, and diagnostic and therapeutic interventions in primary care. In follow-up, the NIVEL set up a permanent representative nationwide network of sentinel practices (LINH), which produces regular monitoring reports on epidemiology, management and organization of general practice in the Netherlands [17].

In the Utrecht region, six general practice groups collaborating with the University Medical Centre started in 1996 a registration network, with the mission to make their routine care data accessible for research [18]. With developing registration systems and training in systematic data recording, the data quality improved over the years, and this academic primary care network became a high-quality cohort for research. In 2016 the network had expanded to 64 practices, and presently consists of routine primary care data of 370,000 enlisted individuals (see Table 1), with 1.38 million consultations annually, and 15–20 years follow-up.

**Table 1:** General characteristics of the Julius General Practitioners Network (JGPN, 2015)

Number of general (group) practices	Total number of patients enlisted	Average number of contacts per day per practice	Total number of patient contacts per year	Mean number of patient contacts per year (range)	
64	371,028	79.9	1384.129	3.7 (0–193)	
Age groups in years	Number of men (%)	Number of women (%)			
0–19	41,405 (11)	39,700 (11)			
20–39	50,722 (14)	60,717 (16)			
40–59	51,256 (14)	51,238 (14)			
> 60	33,965 (10)	40,845 (11)			
Total	177,348 (48)	192,500 (52)			
Top five prescribed drugs according to the Anatomic-Therapeutic Codes (ATC) for medication	Number of prescriptions (% of total)	Number of patients (% of total)	Mean number of prescriptions per patient	Number of patients with just one prescription (%)	Percentage of patients in the Netherlands with the prescription [48]
Antacids (PPI’s, H2-antagonists, and antacids) (A02B)	164,293 (7)	39,613 (11)	4,1	12,619 (32)	16
Statins and other cholesterol lowering drugs (C10A)	134,352 (5)	26,24 (7)	5,1	3183 (12)	11
Antithrombotics (Vitamin K antagonists, NOACs, and anti-platelets) (B01A)	123,209 (5)	22,195 (6)	5,6	3207 (15)	10
Beta-blockers (C07A)	105,737 (4)	21,413 (6)	4,9	3689 (17)	9
Antidepressants (N06A)	100,883 (4)	18,046 (5)	5,6	3048 (17)	Below top 10
Top five diagnoses according to the International Code Primary Care (ICPC) for diagnoses	Number of contacts with such an ICPC-labelled diagnosis (number of new/relapsed episodes)	Percentage of total number of contacts with an ICPC-code (%)	Number of patients (% of total)	Mean number of contacts per patient (minimum-maximum)	Percentage of patients in the Netherlands with the ICPC-code [49]
Hypertension (K86, K87)	52,508 (2708)	3.9	18,445 (5)	2.8 (1–29)	4
Type 1 and 2 Diabetes Mellitus (T90)	51,194 (11,547)	3.8	11,283 (3)	4.5 (1–79)	2
Cystitis/urinary tract infection (U71)	28,834 (19,495)	2.1	12,473 (3)	2.3 (1–35)	3
Cough (R05)	25,487 (22,794)	1.9	16,966 (5)	1.5 (1–15)	2
Upper respiratory tract infection (R74)	23,579 (22,330)	1.7	17,580 (5)	1.3 (1–13)	2

In this paper we describe the organisation and data content of the Julius General Practitioners’ Network (JGPN), the different types of research that have been performed with the data and the potential of the networks for future innovation and how the data can be supportive in education in general practice.

## Methods

### Aims and objectives of the JGPN

The main aims of JGPN are to facilitate clinically relevant research, the development of health care innovations and to support quality management by using routine care data. The network infrastructure brings together routine care registration data from all participating practices. After obtaining consent from a scientific board, the anonymised database can be used for scientific research. The network can generate key indicators of clinical performance of participating practices that can be benchmarked with the rest of the network. The network also supports innovative disease management programs, such as panel management of frail elderly and early identification of patients at risk of depression and cardiovascular disease. In the near future, the network data will be used to monitor the educational progress of GP trainees working in the network practices.

### Organisation and infrastructure

#### Demographics

The current composition of the patient database, and the geographical distribution of the participating practices in urban and (semi) rural regions make the JGPN population representative for the Dutch population. Gender, mean age, and age distribution of patients is comparable to the Dutch population (47.9% versus 49.5% males, 39.5 vs. 41.3 years; Table 1). The number of participating female GPs is higher than the Dutch average (60% vs. 44%), as is the number of group practices in JGPN (76% vs. 33% national average). Median follow-up time in the present JGPN is 6.4 years (IQR 2.4–14.1), with a median follow-up time per practice (*N* = 67) of 8.6 years (IQR 7.4–11.8). Minimum follow-up time is 0 years, maximum 87.8 years. The mean loss to follow-up in the network over the years is 5.3% annually, mainly caused by moving as a result of enlistment to another GP who is not participating in the JGPN network. In 2015, the average number of diagnoses and prescriptions per patient was 2.3 (range 0–45), and 2.5 (range 0–96), respectively. Patients contacted their GP on average 3.7 times per year (range 0–193), with 30% of patients not visiting their GP.

#### The data

The JGPN-database contains structured information of each patient-doctor consultation in the participating practices. This makes it possible to follow the ´health career’ of the individual patient. All contacts are registered according to a systematic format with information on symptoms, signs, diagnostic test results, diagnosis, and patient management, including drug prescription and referral to hospital specialists. Diagnoses are entered according to ICPC coding, hospital referrals are coded according to specialist and prescribed medication (including dose) is entered in ATC (anatomical therapeutic coding). In addition, GP consultations are grouped in episodes, i.e. a series of consultations related to a single reason for encounter (a symptom or a diagnosis). In 2015, for example, the database contained 11,547 new episodes of diabetes mellitus and 22,330 new or recurrent episodes of upper respiratory tract infections (Table 1). Return letters from hospital specialists are received electronically, and linked manually by the GP to the ICPC coded episodes in the EMR. Frequently GPs copy paste a summary and conclusions of letters in free text boxes of the EMR.” Every three months the database is uploaded with the data extractions from the participating practices.

#### Data quality

In general, completeness and accuracy of routine databases depend on the systematic registration of the different steps in the consultation [19, 20].^.^ To optimize and synchronize this coding process, the JGPN management has organised coding training sessions for participating GPs from the beginning [21]. This has resulted in more systematic data registration. Nowadays, the software support is such that entering an ICPC code for every episode is mandatory in most EMRs [9, 22]. As a result, the JGPN database represents a complete and increasingly accurate reflection of the morbidity, follow-up and medical management of the enlisted patient population. The database seems to produce reliable quantitative estimates of symptom incidence, disease prevalence, referral and prescription rates, but also qualitative data on the reason for encounter and patient presentation [23–28].

#### Enriching the database by data linkage

To widen the scope of the individual health data, the JGPN database can be linked to other data sources. Specialist data can be accessed from the databases of hospitals or insurance companies. In the latter database, all secondary care interventions are stored, coded through so called ‘diagnostic therapeutic combinations (DBC) codes. In addition, linkage to national disease or mortality databases like the regional primary care laboratory, the National Cancer Registry or the National Mortality Registry, generates important complimentary sources of information [29]. Technically, data linkage with JGPN is done through a Trusted Third Party (TTP) construction using anonymous pseudo-identification, safeguarding that the patients’ identity is not disclosed to researchers. In the past JGPN data were successfully linked to a regional psychiatric database to assess potential association between somatoform disorders, infectious diseases and antibiotic prescriptions [30]. Sollie et al., managed to validate the cancer diagnoses in JGPN through linkage with the National Cancer Registry [31]. In the future, a virtual network may be constructed in which databases of daily patient encounters of different health organisations, including JGPN, will be connected, thus providing a regional data warehouse for research and quality monitoring.

#### Privacy issues

The JGPN is subject to Dutch privacy law. The law on the medical consultation (Dutch: WGBO) states that the use of medical data for scientific research is allowed provided that i) results cannot be traced to individual patients, ii) and people are adequately informed beforehand allowing them an opt out option. As a consequence, all GPs participating in JGPN inform their patients by practice flyers and/or on their internet site about the anonymous use of their medical records for research purposes. Patients may opt out, and their routine care data will not be used for the JGPN database (opt-out regulation). The law on medical research (Dutch: WMO) states that medical research on patients requires individual consent if an intervention takes place. Research in JGPN is observational, without an intervention, and enlisted patients are not individually approached for participation. Therefore, the Medical Ethics Committees in the Netherlands do not rank such research as subject to the WMO conditions, but researcher need to confirm to the privacy legislation. The complete anonymised dataset is stored safely, and only copies of parts of it are delivered to researchers after they have requested it and JGPN has agreed on executing their research proposal. Such data sets are made only available under the strict condition of anonymity, and researchers have to give written consent to destruct the data at the end of their study. Only under very strict conditions free text is made available to researchers, after free text fields are anonymised by the computer program TM7 that is especially developed for this purpose [32]. In the data linkage process privacy is ensured by the so-called pseudonymisation procedure; linkage codes are destroyed after successful data linkage.

#### Governance

All participating GP practices have a longstanding relationship with the network. In return for their willingness to participate, they receive a small annual fee or benchmark information about their practice of the listed patient population and their medical management. The operational process of the network is run by a small team consisting of a coordinating and operational manager, a data-manager and secretarial support. The use of the database for research is monitored by a steering committee, consisting of representatives of the participating GPs in the network, the operational manager, and supplemented by research advisors of the Julius Centre. As yet, patients are not included on the steering committee. This steering committee also assesses requests for data submitted by researchers following a standard procedure. Research proposals should meet pre-set criteria, including sufficient quality of methodology, clinical relevance, and acceptable burden for practices and patients of JGPN. After consent of the steering committee and signing of the JGPN contract, data management of the JGPN prepares the requested dataset. Researchers pay a fee for each dataset, and thus contribute to the maintenance costs of the JGPN dataset. Most of the running costs are covered by institutional sponsoring of the University Medical Centre Utrecht.

## Results

### Examples of research performed in the JGPN database

In the past 10 years the JGPN database has been used by researchers of the UMC Utrecht as well as by external research groups, which has resulted in 165 research projects and 105 peer reviewed publications. Data have been used for descriptive research and observational studies, such as trends in antibiotic prescriptions, but also for other types of epidemiological research [33–35].

#### Etiologic studies

In etiological research, the causal relationship between a single determinant (i.e. risk factor) and a disease (or outcome) is assessed, but without randomisation the association is distorted by confounders. The most important potential confounders registered in the JGPN database include age, gender, lifestyle, socio-economic status, and co-morbidities. Additional patient characteristics can be obtained through linkage with other databases or through the use of proxy indicators. With supplementary data acquisition etiologic research is possible within the JGPN database.

##### Example of etiological research in the JGPN database

Rutten et al. assessed the long-term effect of beta-blocker use on survival and exacerbation in patients with COPD. After correction for multiple (potential) confounders, the adjusted hazard ratios (HRs) for all-cause mortality and exacerbations were calculated with both propensity scores and Cox regression analyses. [36].

#### Diagnostic studies

Diagnostic studies usually aim to determine the diagnostic value of tests in patients suspected of a certain disease. For this type of research all suspected cases should have undergone the test(s) under investigation, but also the reference test to establish the disease (the outcome), without work-up bias. This is usually not the case in routine care data. Routine care data can however be used to identify potentially useful determinants that may be part of a diagnostic prediction model derived form a cohort with enough necessary information to perform such prediction research.

##### Examples of diagnostic research in the JGPN database

Van de Pol et al. investigated the determinants of referral for recurrent respiratory tract infections (RTI) in young children aged between zero and two years old [37]. In the study of Van Mourik et al eighteen of the JGPN practices participated. In this diagnostic cluster randomized trial opportunistic screening of 1249 frail elderly with reduced exercise tolerance was compared to care as usual for the detection and the following management of newly diagnosed heart failure and COPD [38].

#### Prognostic studies

Prognostic research focusses on prediction, answering the question; ‘which variables predict an outcome such as mortality in patients with a certain disease, irrespective of a possible causal relation [34]. Consequently, confounding is not an issue. These studies are usually performed within a well-defined cohort of patients followed for a period of time to facilitate development of disease. It requires the presence of data of the patient’s condition and of potential determinants, like ICPC-coded morbidity.

##### Example of prognostic research in the JGPN

Bertens et al. developed a prediction model for exacerbations in patients with COPD [39].

#### Intervention studies

Randomized controlled trials (RCTs) are difficult to perform within a cohort of observational routine care data, but as an alternative, a case-control design could be used to evaluate an intervention. Patients cannot be included through randomisation, but sampling techniques can be applied to limit selection bias while selecting controls. In addition, quasi-experimental designs can be used to evaluate interventions, including pre-post comparisons or stepped wedge designs for introduction of innovations in a ‘real life’ setting. To validate the effect, information on potential confounders is needed, otherwise, the validity of the outcomes of these types of studies is hampered.

##### Example of intervention research in the JGPN

Venekamp et al. conducted a study in which a recent update of a primary care guideline on acute rhino sinusitis (ARS) was evaluated. The judicious use of antibiotics by consultation and prescription rates before and after the introduction of the guideline were compared [40].

### Quality of care support and development of health care innovations using the JGPN

Routine primary care data also provide the opportunity for quality support and evaluation of health care innovations. For these ‘interventions’ it is essential the GP receives feedback information on patient level. To enable this, data need to be de-anonymised. The patient’s own GP is the only person having the key to identify his/her patients again.

#### Patient selections for ‘patients at risk’

The principle of ‘panel management’ is that routine care data are used to preselect (symptomatic or asymptomatic) people at risk of a disease. Selected high-risk patients may subsequently be approached by the GP for preventive interventions. The selection frequently requires a priori prognostic research to identify which combination of risk factors optimally predicts the development of a disease [41]. Once the risk profile is identified it can be applied to the JGPN dataset, and participating practices subsequently receive a list of high-risk patients that need to be approached for follow-up actions. In this way, “real time” monitoring and feedback can be offered within the JGPN network.

##### Example of selection of patients at risk management in the JGPN

In the U profit study Drubbel et al. developed a frailty index (FI) for elderly patients that calculates the patient’s risk of frailty based on the patient’s health characteristics and health care consumption. To predict adverse health outcomes, survival curves were constructed and hazard ratios estimated. The FI for all elderly patients in the JGPN were calculated, and patients at risk were reported to the GP, who then could actively approach the patient for care interventions [42].

#### Support in individual patient management

JGPN can also be used to support daily clinical practice by adding (anonymously) new clinical information to the medical files of patients. The researcher may review the routine care data of the GP and (re)calculate the reliability of a particular diagnosis based on the characteristics and clinical data of a patient. Confirmation or cancellation of the diagnosis or new diagnosis is returned (in encrypted form) to the practice to enrich the GPs’ registration and to upgrade the patients’ records.

##### Example of individual patient management in the JGPN

Van Doorn cs. were focussing on the management of atrial fibrillation (AF). Patients with an ICPC code K78 ‘atrial fibrillation/flutter were checked for a confirmatory 12-lead electrocardiogram or heart rhythm registration, and the CHA2DS2-VASc risk score was calculated in every case with confirmed AF. This information was fed back to the GPs. With this information, the GP could re-consider the antithrombotic management of the patients with AF [43].

#### Quality evaluation programs on population level

The JGPN database can also be used for the evaluation of new disease managing care programmes. For example, the introduction of a new guideline may be evaluated by comparing pre and post outcomes such as referral patterns or prescription trends. To select a patient cohort that receives the guideline or managed care programme, arrangements must be made with the GPs concerned. In this type of study the effect on the outcome must be defined by the intervention components, after control for possible confounders such as practice variability. Studies monitoring antibiotic prescriptions are striking examples, such as the study evaluating the introduction of an update of the guideline on acute rhino sinusitis [40].

##### Example of quality evaluation in the JGPN

Van den Broek d’Obrenan described the antibiotic management of infectious diseases in the JGPN. During 2007–2010 all contacts for infections and the rate and choice of antibiotics in 45 practices were analysed [44].

### The use of the JGPN data for educational purposes in GP specialty training

If GP trainees are trained in one of the practices participating in JGPN the routine care data can be used for assessing educational progress by monitoring clinical performance. An important condition is that trainees have their own login code to register their patient contacts in the electronic medical files. Analysing these registrations provides excellent opportunities for monitoring the patient’s mix that GP trainees see, as well as their clinical performance, and trainees can evaluate their performance with their supervisors [45]. The medical management decisions of the trainee can be evaluated against key indicators of professional guidelines. Kortekaas et al. developed a program of key indicators for 27 clinical practice guidelines [46]. Compliance with these indicators can be evaluated using data extracted from the JGPN routine care registration. This system needs further refinement but can provide supportive feedback reports for trainees and their supervisors during the GP specialty training [47].

## Discussion

Routine-care primary care databases such as the JGPN offer excellent potential for different types of clinical research. In addition, the database can be used for feedback and monitoring purposes in support of quality of care programs in daily care. In future the dataset will also be used for educational purposes, to monitor the clinical performance of GP trainees and medical students in primary care practice. Important assets of the JGPN are the size of the data set, the length of the follow-up, the representativeness of its population, and the variety of clinical information that is registered. Much effort has been put in uniformity of the registration of the routine care, resulting in a high accuracy of the data over the years.

### Comparison with other data networks

Many primary care routine datasets exist; most are used only for research purposes. In the Netherlands, for example, all academic universities have such a (research) network. In the UK the General Practice Research Database is focused on facilitating larger scale observational research in primary care. Other networks have surveillance or monitoring purpose, such as the LINH sentinel network of NIVEL in the Netherlands that provides representative (monitoring) data on morbidity and clinical management in primary care. The content and the quality of the data vary between different databases. Some networks only store the coded information, and do not extract the qualitative text data, laboratory results, or the referral letters. The quality mainly depends upon the uniformity in coding, the training that participating GPs received, and the frequency of extractions. Some networks provide extensive training sessions, while others largely depend on routine data entry in the participating practices. The JGPN offers several advantages over other networks: it stores information on all aspects of clinical care; qualitative textual information on reason for encounter, diagnostic data, ICPC coded diagnoses and ATC coded prescription data, and referrals and return letters from specialists. Participating GPs are used to working with a structured coding program. The JGPN is based on a long-standing collaboration between regional participants and the academic department, and the practices actively participate in the management and exploitation of the database. Finally, the JGPN dataset is used for various objectives: not only for academic research, but also for regional surveillance, managed care programs and quality benchmarking purposes as well for educational purposes. This supports the feeling of joint ownership and creates a ‘win-win’ with participating practices.

### Limitations of the database

The JGPN database contains observational data, of the consultations that were registered by the GP, depending on the interpretation of the consultations by the GP and the way it was registered by the GP. This is also the limitation of the dataset. Because of the anonymous character, it is not possible to go back to the patient in order to collect additional information. Additional information becomes available only if the patient reappears on consultation, or if data are linked to other sources. Missing data for specific research questions are not retrievable.

A second limitation of the data is that follow-up may be interrupted by both moving or by death. With the removal of patients from the database, the distinction between those who moved or those who passed away is no longer traceable. Through proxy indicators on morbidity or through linkage to the official death registry (CBS) this limitation can often be overcome.

A third limitation is that the detailed data of the electronic chronic disease management programs, that many practice have introduced to monitor their patients with type 2 diabetes, chronic obstructive pulmonary disease, and cardiovascular risk management, were initially not automatically transferred to the routine care registration. Thus, these data were not available in JGPN. In recent years however, key indicators of chronic disease management programs are automatically copied to the routine care registration.

Finally, data extraction takes place only four times a year, so the data are not always up to date. Given the progress in data capturing techniques, however, a system of real-time monitoring should be realistic in the future.

### Suitability for research; pros and cons

The longitudinal character of the database provides the opportunity to perform observational analyses in different designs. Etiological studies can be achieved by splitting up the cohort and compare the positive and negative study-arm adjusting for the potential confounders. In addition, the database is large enough to analyse matched groups who are matched on important characteristics. The presence of additional determinants such as disease history, signs and test results make the data also attractive for *prognostic studies*. Diagnostic studies are more difficult to perform as test results are incompletely entered in the database. This can be overcome by linking individual data from laboratory databases. Interventions can be evaluated, but only in observational comparisons, with quasi experimental designs. This is particularly effective in the evaluation of healthcare innovations or guidelines after introduction on population level.

One could argue that JGPN contains insufficient performance indicators for monitoring quality of care, since GP practices vary in the level of detail of information that is entered in the routine care registration. As a result, indicators of quality of care cannot always adequately be generated. These limitations, however, can be overcome in two ways. First, many clinical outcomes can be estimated by the use of proxy indicators, such as referrals (for treatment), or medication (for diagnosis). These proxy indicators have intrinsic limitations because of, for example, inter-physician variation in referrals or in individual health policies. Alternatively, clinical information can be enriched by linkage to other databases, such as those from hospitals, insurance companies or from disease-specific registries. Even when a deterministic or probabilistic linkage would result in a number of unidentified individuals, the number of patients in the JGPN is large enough to have adequate statistical power.

### Future challenges

The main future challenges for JGPN are the development of a regional data warehouse, the need to adequately address the medical ethical legislation for large-scale routine care data collection and the need to safeguard the commitment of partners such as GPs and patients. JGPN was initially setup as a mono disciplinary registration network. However, for optimal use of the data in future, structural linkage to other sources is essential to meet the ever- increasing demands of researchers, to continuously upgrade the quality of the data and to broaden the scope of the database. Recently, linkage to laboratory data, disease registries, and hospital data proved successful, but time consuming. In future, real-time linkage through a virtual regional data warehouse, connecting all relevant data sources in the region, should overcome these limitations.

Legislation around medical databases is becoming more strict, to protect the privacy of patients and medical professionals. Many suggest that individual Informed consent is required for optimal protection, but experience has learnt that only a minority of patients do respond to requests for research participation. This would threaten both the size of the database as well as its representativeness. The present opt out procedure, with optimal information provision in the practices, a register of patients that refuse to participate, and shared governance over the data, adequately safeguards ethical use of the data. Ultimately, it is the patient, and not the professional or the researcher, that decides on the use of the individual health data, even though they are anonymised. Therefore not only researchers and GPs, but also patients should be actively involved in the JGPN steering committee. Patient participation is therefore one of the challenges for the near future.

## Conclusions

Routine-care primary care databases such as the JGPN offer excellent potential for different types of clinical research. Moreover, such databases can be used to support quality management in participating practices, thus optimizing individual patient care. This secures the balance between academic interest, and value for the participating GPs and patients, stimulating the concept of joint ownership, and turning a database into a solid instrument in regional health care developments.
